# Keratoconus with two consecutive re-emergences: a case report

**DOI:** 10.1186/s12886-025-03877-4

**Published:** 2025-02-04

**Authors:** Peicheng Zhang, Yanchao Wu, Lixiao Ma, Dong Wang

**Affiliations:** Hebei Provincial Key Laboratory of Ophthalmology, Hebei Provincial Clinical Medical Research Center for Ocular Diseases, Hebei Eye Hospital, Xiangdu District, 399 Quanbei East Street, OphthalmologyXingtai, 054001 China

**Keywords:** Keratoconus, Recurrent keratoconus, Deep anterior lamellar keratoplasty, Chronic allergic conjunctivitis, Eye-rubbing

## Abstract

**Background:**

Keratoconus is a common disease in clinical practice, and deep anterior lamellar keratoplasty(DALK) is a conventional treatment for keratoconus, which is effective and not easy to recur after surgery.

**Case presentation:**

The author presents a rare case report involving a 21-year-old patient with Consecutive re-emergences of Keratoconus. He underwent DALK for keratoconus in his left eye in 2012 and experienced re-emergence 3 years later, followed by a second deep lamellar keratoplasty in the same year. In 2019, the patient experienced re-emergence of keratoconus and underwent a third deep lamellar keratoplasty in the same eye. Genetic testing was performed, and no abnormal genes were identified. Postoperative follow-up emphasized the management of chronic allergic conjunctivitis (CAC) and the correction of eye-rubbing habits. To date, he has not experienced any further re-emergence.

**Conclusions:**

Keratoconus is a complex disorder with a multifaceted etiology and pathogenesis, including genetic, environmental, biomechanical, and cellular factors. Its treatment usually requires multiple considerations, including the choice of surgical methods, anti-inflammatory therapy, and correction of eye rubbing habits to guide patients for subsequent treatment interventions.

## Background

Keratoconus is a non-infectious ectatic corneal degeneration. Traditionally considered a noninflammatory disease, recent evidence suggests an association with ocular inflammation. Previous literature reports occasionally remind us that keratoconus may recur after corneal transplantation. Abelson reported 1 case respectively, and thought that the stromal cells and endothelial cells of the graft bed would invade the graft tissue, thus causing the pathological changes of keratoconus [1]. Interestingly, the latency of re-emergence is considerably shorter after lamellar keratoplasty (average 3–4 years) compared to penetrating keratoplasty (average 19 years) [2]. This supports the clinical hypothesis that re-emergences stem from the underlying pathology in the non-excised corneal tissue. Steven believes that the re-emergence of keratoconus is due to chronic mechanical damage to the cornea [3]. For the first time, we reported a case of consecutive keratoconus recurrence after two consecutive deep anterior lamellar keratoplasty(DALK). Our findings suggest that recurrent keratoconus may be a long-term complication of the eye-rubbing habit caused by chronic allergic conjunctivitis (CAC).

## Case presentation

**Fig. 1:**
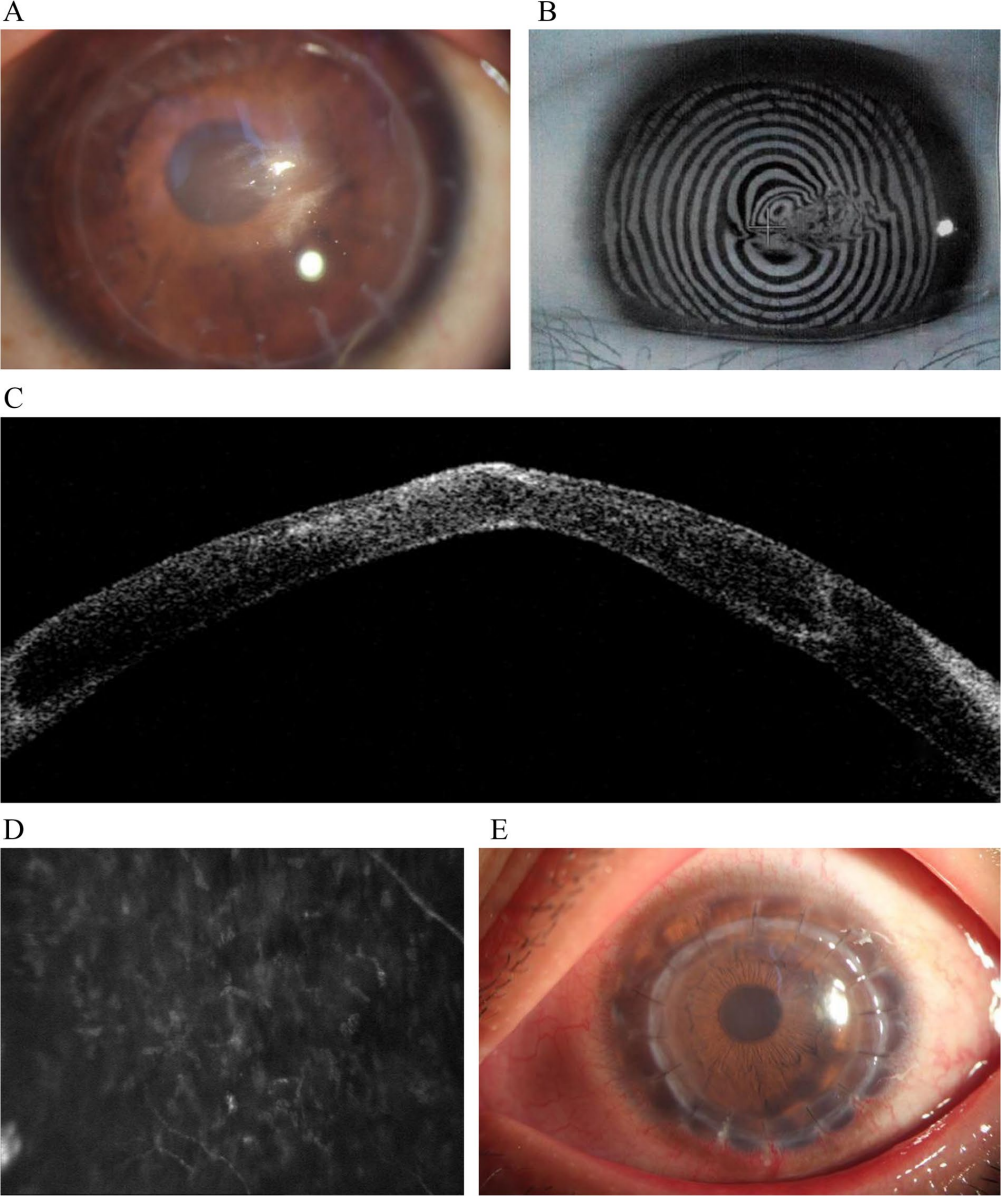
**A** The central area of the corneal graft was conical and slightly thinned, with the apex located slightly temporal to the center. **B** Severe anterior protrusion and stromal scarring, resulting in an inability to obtain images. **C** The central cornea was thinned and anteriorly protruded, with stromal opacity located in the superficial stroma. **D** The distribution of anterior stromal cells was uneven, with some activated cells exhibiting nuclear fusion and connection. The dark areas may represent sites of stromal lamellar disruption. **E** Best-corrected visual acuity after the second DALK is 20/33 (+ 2.00DC × 75)

**Fig. 2:**
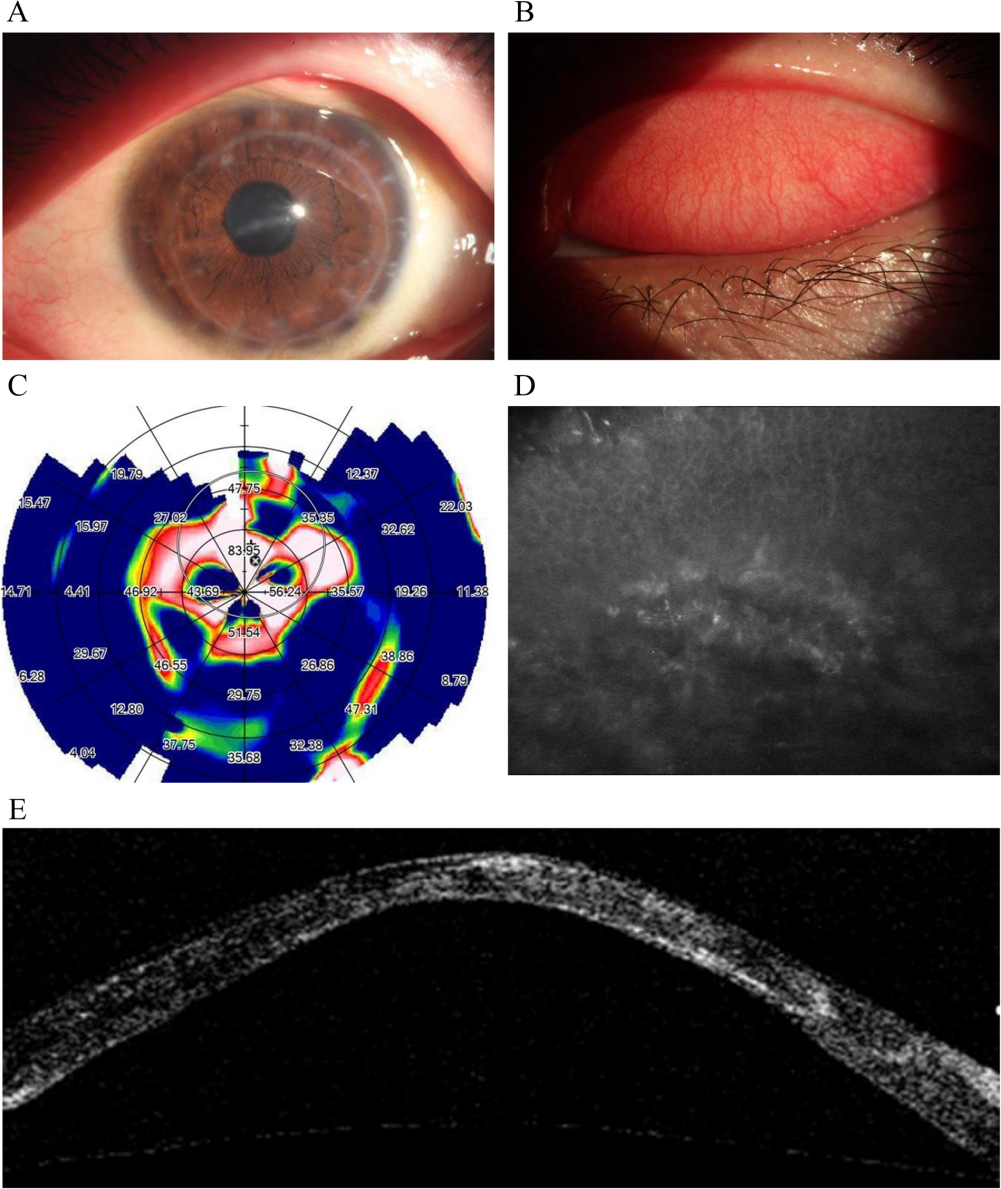
**A** A horizontal grey-white stromal scar measuring approximately 0.5 × 2.5 mm was seen at the top of the cone, along with a Fleischer ring measuring approximately 4 × 5 mm. **B** Moderate congestion was observed in the palpebral conjunctiva of the upper eyelid, with numerous papillary projections present. **C** The cornea exhibited high irregular astigmatism, with a maximum corneal curvature of 83.95D. **D** The stromal cell structure in the superficial stroma was disorganized and in an activated state. **E** The central area of the corneal graft exhibited stromal thinning and anterior protrusion

In 2019, a 21-year-old male patient with a history of corneal transplantation underwent a routine ophthalmologic examination at a tertiary eye center, complaining of decreased vision in his left eye for nearly one and a half months. In June 2012, the patient underwent DALK in the left eye for keratoconus, during which the big-bubble technique was employed. A corneal graft with a diametr of 8.0 mm was sutured onto an 8.0 mm corneal bed. In June 2015, he presented with a 3-month history of decreased vision in the left eye. Slit-lamp examination revealed central corneal graft protrusion and slight thinning in the left eye, with the apex located slightly temporal to the center. A grayish-white superficial stromal scar measuring approximately 1 × 2 mm was visible at the apex, and a Fleischer ring measuring approximately 3 × 4.5 mm was present (Fig. 1A). The left eye had high irregular astigmatism (Fig. 1B), and anterior segment optical coherence tomography (OCT) showed that the opacity was located in the superficial stroma (Fig. 1C). Confocal microscopy of the anterior stroma showed uneven distribution of stromal cells (Fig. 1D). On June 18, 2015, due to recurrent keratoconus in the left eye, DALK was performed again. During surgery, the graft was bluntly separated along the original graft-host junction and sutured with a graft of equal size to the host bed (Fig. 1E). In 2019, the patient presented again with decreased vision in the left eye. Examination revealed that the central area of the left corneal graft was conical and convex, with slight thinning (Fig. 2A). Upon everting the upper eyelid, moderate congestion was observed, and the palpebral conjunctiva had numerous papillary projections (Fig. 2B). At this visit, corneal topography, confocal microscopy, anterior segment OCT, and histology of tissue were performed to assist in diagnosis (Fig. 2C - E). The patient underwent genetic testing by a third-party laboratory which analyzed exons and adjacent splice regions of genes contained in an ophthalmic genetic disease panel and focused on known causative genes for related phenotypes and genetic disorders such as keratoconus; no matching variants were identified. A diagnosis of recurrent keratoconus and CAC were made. In August 2019, the patient underwent another DALK in the same eye. A corneal graft with a diameter of 8.25 mm was sutured onto an 8.25 mm corneal bed. Postoperative follow-up emphasized the management of CAC and the correction of eye-rubbing habits. No signs of re-emergence were observed at the April 2024 follow-up.

## Discussion and conclusions

The etiology of keratoconus remains incompletely understood and continues to be a subject of discussion due to the potential involvement of various pathways: genetic, epigenetic, biochemical, inflammatory, and mechanical. The act of repetitive and prolonged eye rubbing is acknowledged as a significant risk factor and catalyst for the progression of keratoconus [4]. The cessation of eye rubbing could potentially aid in halting the progression of keratoconus [5]. There is some debate about the direct pathogenic role of eye rubbing, so we still need direct evidence that mechanical factors may be an independent pathogenic factor in the pathogenesis of cone cornea.

In the absence of any ocular disease, the duration of rubbing was generally less than 5 s, less than 15 s in patients with atopic eye disease or infectious eye disease, and between 10 to 180 s in patients with keratoconus [6]. The mechanical force caused by rubbing the eyes with force can stretch the thin and weak areas of the cornea, causing the stressed parts of the cornea to flatten or become concave, while the unstressed parts become steep, leading to distortion of the keratin fibrils. At the same time, the normal production and distribution of collagen are disrupted by the crushing force of eye rubbing and the swelling force of increased eye pressure, ultimately affecting the biomechanical properties of the cornea [7]. Rubbing the eyes can lead to the release of inflammatory mediators such as MMP-13 and cytokines such as IL-6 and TNF-a in the cornea, resulting in the weakening and remodeling of corneal tissue [8]. CAC and eye rubbing may have a synergistic effect. Example, during the active phase of vernal keratoconjunctivitis (VKC), eosinophils release toxic enzymes such as eosinophil major basic protein, eosinophilic cationic protein, and eosinophil peroxidase during degranulation. These enzymes are cytotoxic to corneal epithelial cells. The destruction of the epithelial barrier activates corneal fibroblasts under the action of matrix metalloproteinases, epidermal growth factor, fibroblast growth factor and transforming growth factor-β1, thus promoting their proliferation [9].

Our case report is the first to document two re-emergences of keratoconus following DALK. Following the third DALK and intensive management of CAC and correction of eye rubbing habits, no signs of re-emergence were observed during follow-up. It is recommended that post-keratoplasty patients be closely monitored for lid conjunctiva during follow-up, with emphasis on management of CAC and correction of eye rubbing habits to prevent such complications.

In conclusion, while the etiology of keratoconus is multifactorial, involving genetic, epigenetic, biochemical, inflammatory, and mechanical pathways, the role of eye rubbing as a significant risk factor and potential catalyst for its progression cannot be overlooked. The mechanical stress from eye rubbing can lead to corneal distortion and disruption of its biomechanical properties, while also triggering the release of inflammatory mediators that weaken and remodel the corneal tissue. Our case report underscores the importance of managing atopic eye conditions and correcting eye rubbing habits, especially in patients who have undergone corneal transplantation, to prevent re-emergence of keratoconus. Close monitoring and proactive management of these factors are crucial in the post-keratoplasty period to ensure long-term stability and success of the procedure.

## Data Availability

No datasets were generated or analysed during the current study.

## Abbreviations

CAC: Chronic allergic conjunctivitis
DALK: Deep anterior lamellar keratoplasty
OCT: Optical coherence tomography
VKC: Vernal keratoconjunctivitis
